# Heat shock protein 70 is associated with duration of cell proliferation in early pod development of soybean

**DOI:** 10.1038/s42003-024-06443-8

**Published:** 2024-06-21

**Authors:** Seiya Tanaka, Yuri Ariyoshi, Takatoshi Taniguchi, Andressa C. S. Nakagawa, Norimitsu Hamaoka, Mari Iwaya-Inoue, Chetphilin Suriyasak, Yushi Ishibashi

**Affiliations:** 1 Faculty of Agriculture, Kyushu University, Fukuoka, 819-0395 Japan; 2 Japan International Research Center for Agricultural Sciences, Tsukuba, 305-8686 Japan

**Keywords:** Plant sciences, Agriculture

## Abstract

Pod is an important organ for seed production in soybean. Pod size varies among soybean cultivars, but the mechanism is largely unknown. Here we reveal one of the factors for pod size regulation. We investigate pod size differences between two cultivars. The longer pod of ‘Tachinagaha’ is due to more cell number than in the short pod of ‘Iyodaizu’. *POD SIZE OF SOYBEAN 8* (*GmPSS8*), a member of the heat shock protein 70 *(HSP70*) family, is identified as a candidate gene for determining pod length in a major QTL for pod length. Expression of *GmPSS8* in pods is higher in ‘Tachinagaha’ than ‘Iyodaizu’ and is highest in early pod development. The difference in expression is the result of an in/del polymorphism　which includes an enhancer motif. Treatment with an HSP70 inhibitor reduces pod length and cell number in the pod. Additionally, shorter pods in *Arabidopsis hsp70-1/-4* double mutant are rescued by overexpression of *GmPSS8*. Our results identify *GmPSS8* as a target gene for pod length, which regulates cell number during early pod development through regulation of transcription in soybean. Our findings provide the mechanisms of pod development and suggest possible strategies enhancing yield potential in soybean.

## Abstract (web summary)

The pod is a crucial organ for soybean seed production. One of the HSP70 family genes is associated with pod size regulation mediated by duration of cell proliferation.

## Introduction

Pods (fruits) protect developing seeds from various stresses. The soybean pod consists of two halves of a single carpel^1,2^. The pod wall accumulates nitrogen and photosynthates, redistributing them to seeds^3,4^, and supplies extra photoassimilates^5^, making soybean pod a crutial organ for seed production.

In soybean, pod development starts after pollination same as other plants. The differentiation of carpels into fruits complete by 6 days after flowering (DAF), except in the sclerenchymatous-fiber, pulp and inter epidermis by 10 days, and cell elongation continues to 16 DAf^6^. Pod elongation stops before cessation of seed growth^7^, and pods physically restricts seed growth^8,9^. Therefore, pod size might play an important role in seed growth restriction. In fact, there is a positive correlation between pod size and seed size^10^. Therefore, pod size might play role as an indirect indicator of seed size for soybean breeding^10,11^,which understanding of pod size regulation might provide a method for manipulation of seed size.

Generally, fruit development starts after pollination, with initial cell proliferation followed by cell expansion^12–14^. Pod size depends on both cell number and cell area^15^. Cell number is critical in determining fruit size in cowpea, rabbiteye blueberry, sweet cherry, peach, olive, and apple^16–21^. Cell area can account for natural variation of fruit size in rapeseed^22^.

Many studies have investigated the genes underlying fruit size. *fw2.2* (*SlCNR*), *fw3.2* (*SlKLUH*), and *SlARF9* control tomato fruit size. *fw2.2* and *SlARF9* negatively regulate cell proliferation during early fruit development: greater transcription results in smaller fruit^23,24^. *fw3.2* positively regulates cell proliferation^25^. In rapeseed, *BnCYP78A9* positively regulates silique length by promoting cell elongation in valves^26^. *BnARF18* regulates cell growth in the silique wall via an auxin-response pathway^24^. Overexpression of *miR160* reduced silique length and decreased expression of *ARF10/16/17*, and thus may negatively regulate cell proliferation via an auxin-response pathway^27^^.^ Thus, fruit size is a complex trait controlled by many genetic factors.

Soybean pod length varies among soybean cultivars and showed high heritability^28^. Crossing two soybean cultivars shows that pod length is affected by the action of additive genes with duplicate epistatic effects^29^. Although molecular mechanisms of the control of fruit growth via modification of cell proliferation or cell expansion have been investigated, the mechanisms underlying pod length regulation in soybean remain largely unknown.

Here, we analyzed differences in pod morphology between two cultivars. To investigate genetic factors responsible for pod growth regulation, we performed QTL analysis to clarify genomic regions controlling pod length. We also conducted genetic screening based on microarray and expression QTL (eQTL) analyses to detect a candidate gene by transcript levels. Moreover, we tested the association with pod length of the target gene by using inhibitor treatment. Finally, we analyzed the function of the gene on silique length in *Arabidopsis* knock-out mutants and complemental lines.

## Results

### Temporal changes in pod length

**Fig. 1:**
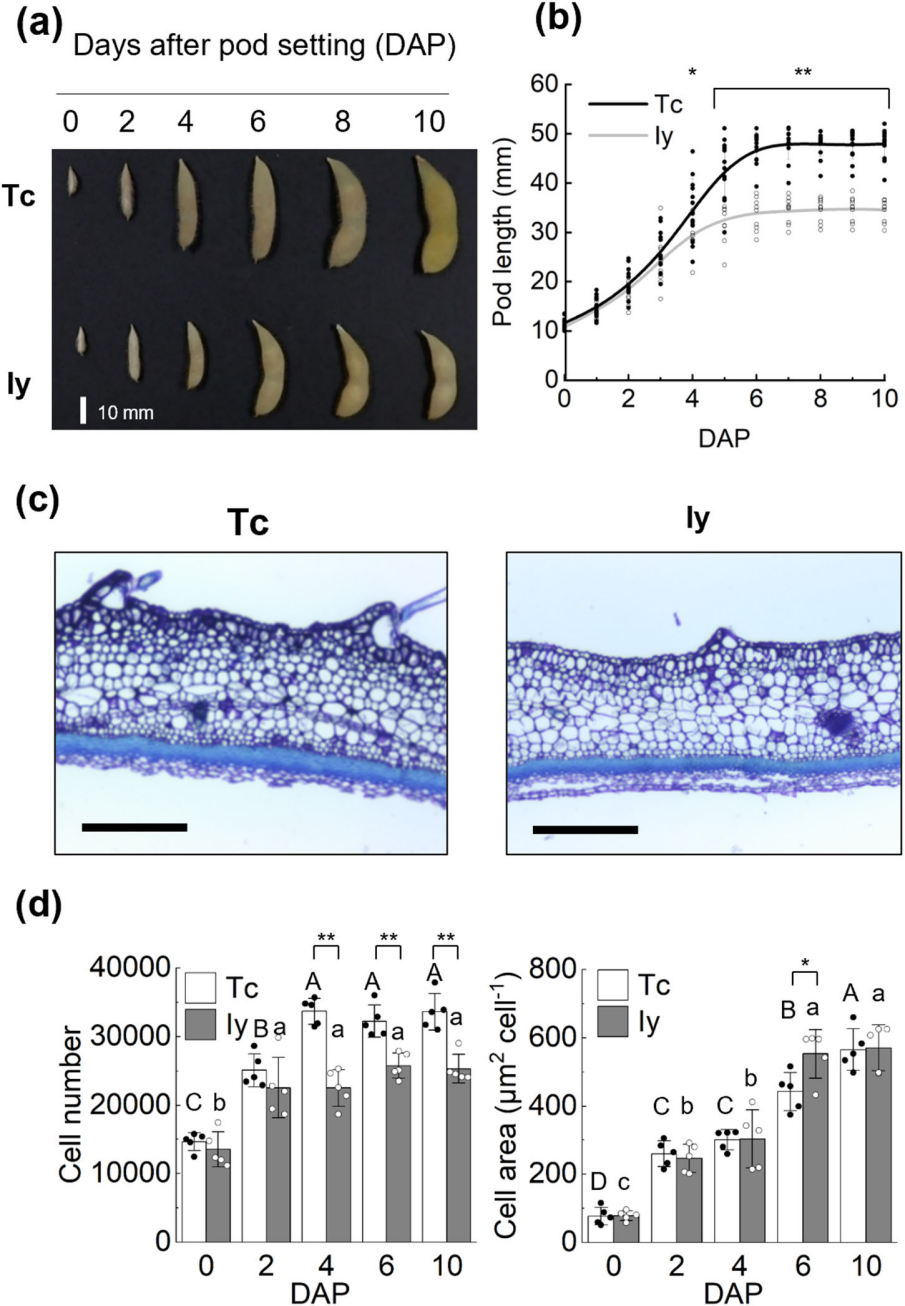
The differences of pod length between “Tachinagaha” (Tc), and “Iyodaizu”; (Iy). **a** A figure showing the growth changes of pod every 2 days from 0 DAP (10 mm-pod) to 10 DAP; scale bar = 10 mm. **b** Changes of pod length between Tc and Iy; *n* = 11. **c** Figures of pod longitudinal section at 10 DAP; Scale bar = 500 µm. **d** Cell number and cell area on longitudinal section of pod at 0, 2, 4, 6, and 10 days after pod setting (after reaching 10 mm-pod, DAP), *n* = 5. Bars with the same letter are not significantly different by Tukey–Kramer method, *P* < 0.05. Significance by Student’s t-test: *P < 0.05, **P < 0.01. Error bars indicate SD.

At 10 days after pod set (DAP) when pods are in the resting stage, pod length was 47.6 mm in “Tachinagaha” (Tc) and 34.6 mm in “Iyodaizu” (Iy). Pod length was significantly higher in Tc than Iy from 4 DAP (Fig. 1a, b).

Organ size depends on both cell number, regulated by cell proliferation, and cell area, regulated by cell expansion (Mizukami 2002). We examined whether the difference in pod length between the cultivars was due to the cell number or cell area of pod. Cell division ceased at 4 DAP in Tc and 2 DAP in Iy. Cell area reached full size at 6 DAP in both cultivars. Cell number was greater in Tc at 4, 6 and 10 DAP than in Iy; cell area did not differ except at 6 DAP. These results suggest that the longer pods of Tc due to the longer cell proliferation activity, not to greater cell expansion (Fig. 1c, d).

### Mapping of QTLs for pod length

**Fig. 2:**
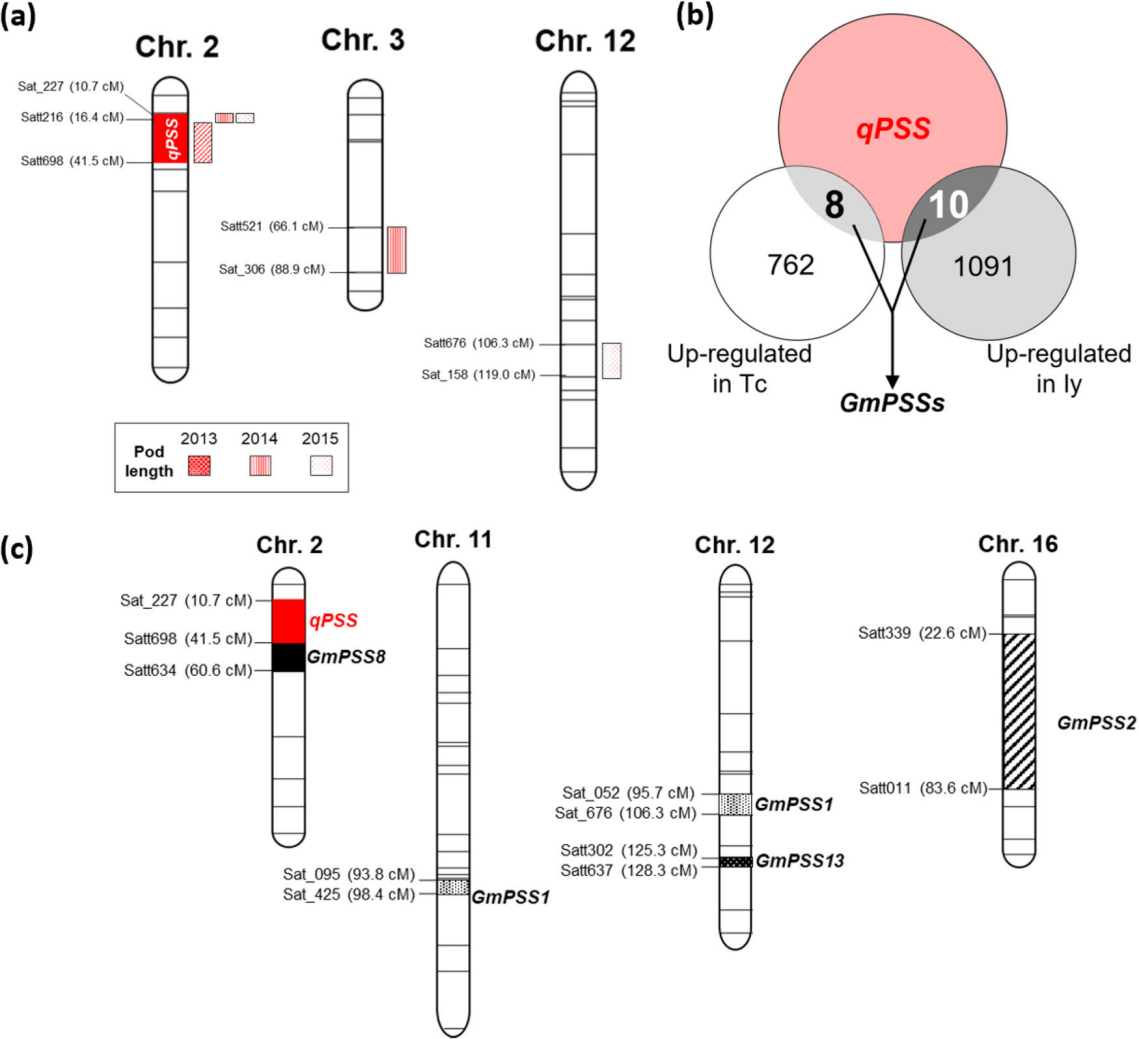
Candidate gene detection by expression QTL (eQTL) analysis. **a** QTL mapping of pod length in 91 Tc × Iy RILs over 3 years. Each colored rectangle indicates the position of a QTL. Details are shown in Table S1. **b** Overlap of differentially expressed genes detected on *qPSS* and genes upregulated in Tc or Iy pods. *GmPSS* annotation is shown in Table 1. **c** Detected eQTLs of *GmPSS1*, *2*, *8*, and *13*.

Pod length averaged 48.5 mm in Tc and 34.2 mm in Iy in 2013, 45.9 and 33.4 mm in 2014, and、51.2 and 37.8 mm in 2015. The pod length of the 91 RILs had a continuous distribution in each year (Supplementary Fig. 1). Significant QTLs for pod length were detected between markers Satt216-Satt698 (16.4-41.5 cM, *R*^*2*^ = 11.38%) region in Chr.2 in 2013, on the Sat_227-Satt216 (10.4-16.4 cM, R^2^ = 9.49%) region in Chr. 2 and Satt521-Sat_306 (66.1-88.9 cM, R^2^ = 14.42%) region in Chr. 3 (R^2^ = 14.42%) in 2014, and on the Sat_227-Satt216 (10.4-16.4 cM, R^2^ = 11.96%) region in Chr. 2 and Satt676-Sat_158 (106.3-119.0, R^2^ = 10.27%) in Chr. 12 in 2015 (Fig. 2a, Supplementary Fig. 1). Only Satt216 on Chr. 2 was identified in all 3 years, suggesting strong association with pod length. Therefore, we defined a QTL region regulating pod length between Sat_227 (10.7 cM) and Satt698 (41.7 cM) on Chr. 2, bracketing Satt216, as *POD SIZE OF SOYBEAN* (*qPSS*). The additive effect of the Tc allele was 0.914 mm in 2013, 0.708 mm in 2014, and 0.995 mm in 2015. (Supplementary Table 1).

### Candidate gene regulating pod growth

To identify the candidate gene for pod growth regulation, we carried out a soybean oligomicroarray analysis of developing pods at 9 DAP. There were 762 genes upregulated in Tc and 1091 genes in Iy (|Z | ≥ 2, Fig. 2b, Supplementary data 1). Of these, 8 genes in Tc and 10 genes in Iy were detected as *GmPSSs*; these genes were designated as *GmPSS1-GmPSS8* and *GmPSS9-GmPSS18*, respectively (Fig. 2b, Table 1). Real-time PCR of their transcript levels showed that *GmPSS1*, *2* and *8* were highly expressed in Tc, and *GmPSS13* was highly expressed in Iy (Supplementary Fig. 2). Thus, *GmPSS1, 2, 8* and *13* are candidate genes involved in pod growth.

**Table. 1:** Gene list *of GmPSSs* detected QTL and microarray analysis

*GmPSSs*	Accession number		Annotation
High expression in “Tachinagaha”			
*GmPSS1*	CD397137.1	*Glyma02g05010*	Cupin domain
*GmPSS2*	CX548460.1	*Glyma02g05170*	O-LINKED N-ACETYLGLUCOSAMINE TRANSFERASE, OGT
*GmPSS3*	BW668140.1	*Glyma02g05480*	N.A.
*GmPSS4*	AI938959.1	*Glyma02g05540*	RIBOSOMAL PROTEIN L2
*GmPSS5*	AW156573.1	*Glyma02g06990*	ALPHA/BETA HYDROLASE RELATED
*GmPSS6*	BE210309.1	*Glyma02g072400*	arabinogalactan protein 14
*GmPSS7*	BM891108.1	*Glyma02g09600*	AP2 domain
*GmPSS8*	BG840055.1	*Glyma02g10320*	HEAT SHOCK PROTEIN 70KDA
High expression in “Iyodaizu”			
*GmPSS9*	BQ742151.1	*Glyma02g03890*	Armadillo/beta-catenin-like repeat
*GmPSS10*	CO979091.1	*Glyma02g04210*	SERINE-THREONINE PROTEIN KINASE, PLANT-TYPE
*GmPSS11*	BE659173.1	*Glyma02g04250*	N.A
*GmPSS12*	BW674267.1	*Glyma02g04930*	F-BOX FAMILY PROTEIN
*GmPSS13*	BI969103.1	*Glyma02g05930*	Peroxidase
*GmPSS14*	BW684714.1	*Glyma02g06610*	Protein of unknown function, DUF607
*GmPSS15*	BW662239.1	*Glyma02g07950*	PROTEIN-L-ISOASPARTATE METHYLTRANSFERASE
*GmPSS16*	CF807784.1	*Glyma02g08835*	AP2 domain
*GmPSS17*	BI971059.1	*Glyma02g09841*	N.A.
*GmPSS18*	CX706471.1	*Glyma02g10380*	RNA AND EXPORT FACTOR BINDING PROTEIN

An eQTL is characterized as *cis-* or *trans*-acting depending on the physical distance from the gene^30^. The eQTL of *GmPSS8* was identified on Chr. 2 (R^2^ = 16.58%) as *cis*-acting eQTL (≤5 Mb upstream or downstream of the gene)^31^, whereas the eQTLs of *GmPSS1*, *2* and *13* were *trans*-acting (>5 Mb away or on a different Chromosome): *GmPSS1* on Chr. 11 (R^2^ = 10.57%) and Chr. 12 (R^2^ = 14.72%), *GmPSS2* on Chr. 16 (R^2^ = 34.8%), and *GmPSS14* on Chr. 12 (R^2^ = 10.38%) (Fig. 2c, Supplementary Table 2). Song et al. ^32^. suggested that a *cis-*acting eQTL is evidence that closely linked regulatory elements directly affect its expression. Therefore, we focused on *GmPSS8* as the candidate gene for pod growth regulation.

### Expression profile of *GmPSS8* in developing pod

**Fig. 3:**
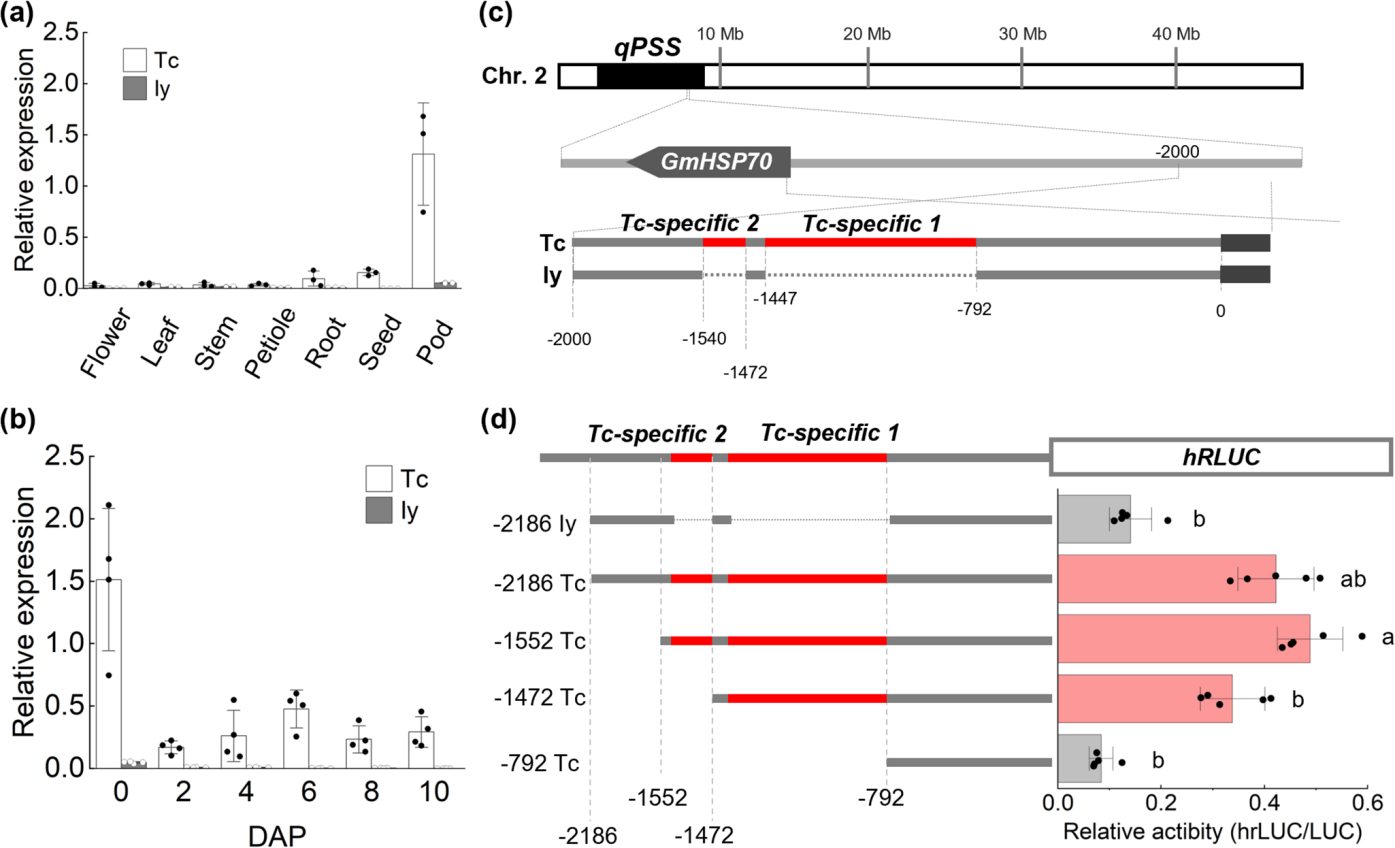
*GmPSS8* expression in Tc and Iy. **a** *GmPSS8* expression in flower, leaf, stem, petiole, and root at flowering, in developing seed (~5 mm), and in 10-mm pod; *n* = 3. **b** Expression pattern of *GmPSS8* in developing pods at indicated number of days after pod set (DAP) of 10-mm pod; *n* = 4. Expression was calculated relative to that of *GmEF1b*. **c** Gene structure and sequence comparison of *GmPSS8* promoter between Tc and Iy. **d** Structure of promoter fragments used for reporter assay and relative activity of reporter gene (*Renilla luciferase*: *hRLUC* / *firefly luciferase*: *fLUC*). Red line, insertion; dotted line, deletion. Bars with the same letter are not significantly different (Tukey–Kramer method; *P* < 0.05; *n* = 4). Sig*n*ificance by Student’s t-test: *P < 0.05, **P < 0.01. Error bars indicate SD.

We investigated the expression of *GmPSS8* in both cultivars. The expression of *GmPSS8* in Tc was 2.2×—367.7× that in Iy in all tissues, notably in pods (Fig. 3a). In developing pods, the expression level of *GmPSS8* was highest at 0 DAP (10 mm) in both cultivars. *GmPSS8* expressed much more in Tc than Iy during all stages of pod development (Fig. 3b).

Microarray analysis annotated *GmPSS8* as “*HEAT SHOCK PROTEIN 70* *kDa*” (*HSP70*) (Table 1). GmPSS8 protein was localized in cytoplasm and nuclear (Supplementary Fig. 3). The amounts of GmPSS8 protein were confirmed in Tc and Iy pods using the anti-cytoplasmic HSP70 antigen to verify protein accumulation during pod development. The amounts of GmPSS8 were higher in Tc than in Iy throughout whole pod development period and tended to decrease as pod development proceeds in both cultivars. Although the highest amount was observed at 2 DAP in Tc and 2.3-fold higher than in Iy at this point, the differences between Tc and Iy become smaller, 1.2-fold higher in Tc than in Iy, at 4 DAP (Supplementary Fig. 4).

Because 27 cytoplasmic HSP70 homologs have been identified in soybean^33^, the expression of *GmPSS8* (*Glyma02g10320*) and the top three highly expressed HSP70 genes (*Glyma03g32850*, *Glyma13g19300*, *Glyma19g35560*) were evaluated in 10 mm-pods. *GmPSS8* and *Glyma03g32850* were higher in Tc than in Iy (30.8 fold and 1.56 fold, respectively). *Glyma13g19300* was higher in Iy than Tc (1.39 fold). *Glyma19g35560* was similar between the two cultivars. *GmPSS8* is greatly different between the Tc and Iy, whereas *Glyma03g32850*, *Glyma13g19300 and Glyma19g35560* were not different between the two cultivars as well as *GmPSS8* (Supplementary Fig. 5), indicating the significance of *GmPSS8* to HSP70 accumulation during pod development. Thus, these results suggested that HSP70 accumulation in early pod development is involved in the differences in pod length in Tc and Iy.

### Transcriptional activity of *GmPSS8* in Tc and Iy

We confirmed no differences of nucleotide sequences affecting amino acid sequences in coding sequences (Supplementary Fig. 6), and the result indicated that the coding region of *GmPSS8* was not involved in differences between Tc and Iy phenotypes. Thus, we examined the promoter region of *GmPSS8* in both cultivars, because the expression of *GmPSS8* was consistently higher in Tc than in Iy. Within the first 2000 bp upstream of the start codon, insertions relative to Iy were detected at 792-1447 and 1472-1540 bp from the start codon of Tc; we named these regions *Tc-specific-1* and *-2* (Fig. 3c, S7). We sought putative cis-acting regulatory elements in both regions in the PlanCARE (http://bioinformatics.psb.ugent.be/webtools/ plantcare.html) and found 15 elements were in *Tc-specific-1* and -*2* elements in *Tc-specific-2*, respectively (Supplementary Table 3). *Tc-specific-1* included a 5”UTR pyrimidine (Py)-rich stretch (Supplementary Fig. 7), annotated as a cis-acting element conferring high transcription levels. To confirm whether *Tc-specific-1* and *-2* play role in enhancing *GmPSS8* expression, we analyzed *GmPSS8* promoter activity by dual-luciferase assay in soybean leaf. Luciferase activities of promoter fragments *−1472 Tc*, *−1552 Tc* and *−2186 Tc* (which include *Tc-specific-1*) were 4.0× to 5.8× that in *−792 Tc* (which does not include *Tc-specific-1*). Those of, *−1552 Tc* and *−2186 Tc*, (which include *Tc-specific-2* region, were 1.45× and 1.25× that in *−1472 Tc*. Thus, *Tc-specific-1* had higher transcriptional activity, whereas the activity of the Iy promoter, *−2186 Iy*, was similar to that of the *−792 Tc* reporter (Fig. 3d). These results suggest that the expression of *GmPSS8* is transcriptionally regulated by *Tc-specific-1*.

### HSP70 inhibitor suppresses the plant growth and pod development in soybean

**Fig. 4:**
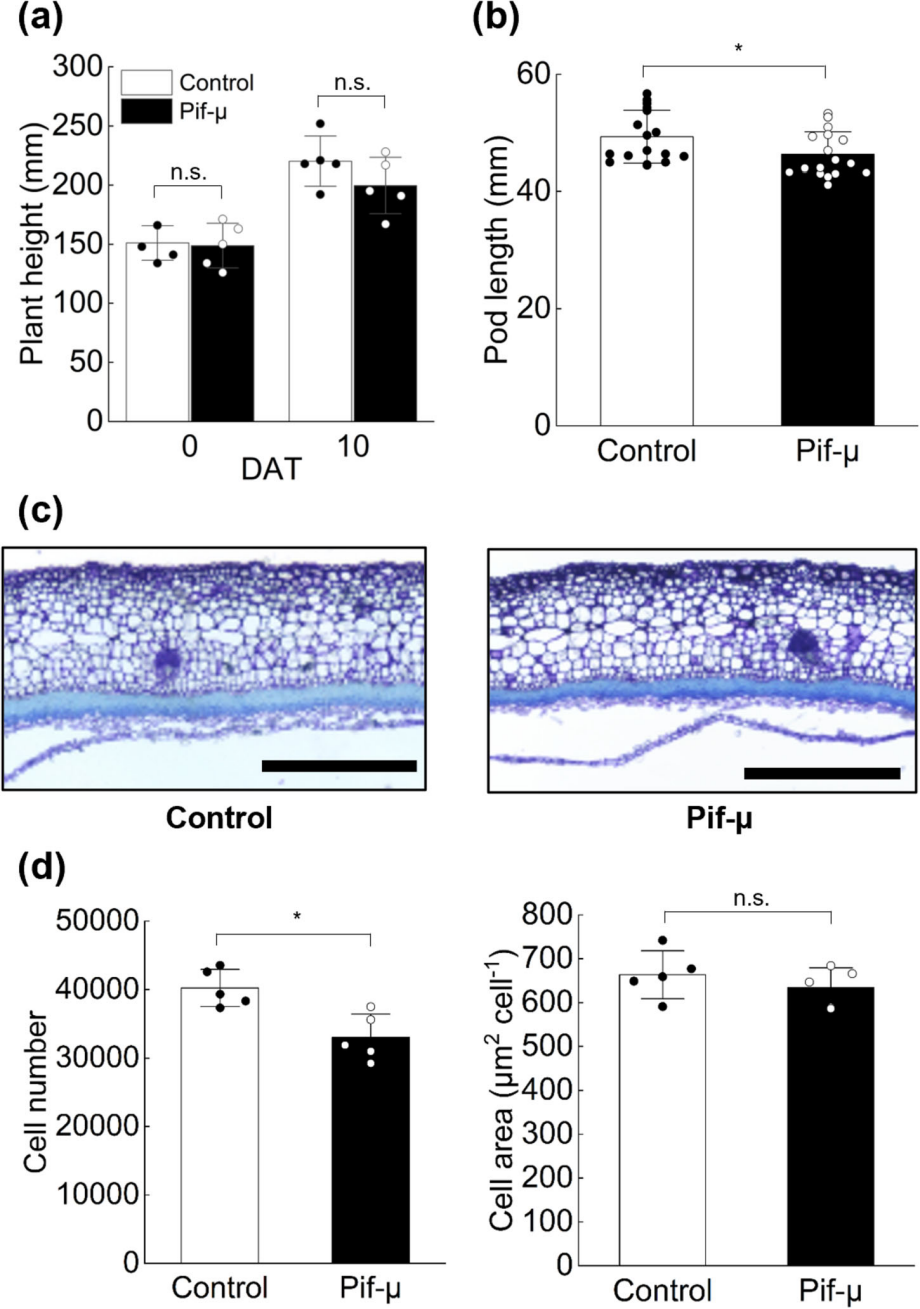
Effects of HSP70 inhibition on vegetative growth and pod growth in Tc. **a** Plant height at 0 and 10 days after treatment (DAT); *n* = 5. **b** Pod length at 12 DAT; *n* = 19. **c** Longitudinal section and **d** cell number and area in longitudinal sections of pod; *n* = 5. Error bars indicate SD. Scale bar = 500 µm. Significance by Student’s *t* test : *P < 0.05, **P < 0.01, n.s.; no significance.

*Arabidopsis hsp70-1/4* double and *hsp70-2/4/5* triple mutants have shortened growth periods, curly and round leaves, twisted petioles, thin stems, and short siliques, suggesting that HSP70 participates in diverse developmental processes^34^. Therefore, we examined the effect of an HSP70 inhibitor, pifithrin-µ (pif-µ), on plant height and pod length in Tc.　In pif-µ-treated plants, despite no significantly change in plant height (Fig. 4a), pod length was significantly decreased compared to that of non-treated plants (Fig. 4b). Thus, we analyzed pod morphology to clarify whether inhibition of pod growth by pif-µ treatment is attributable to cell number or cell size. The pif-µ treatment decreased cell number, but not cell area, (Fig. 4c, d). Thus, HSP70 is associated with pod growth regulating cell proliferation.

### *GmPSS8* rescues silique phenotypes in *Arabidopsis hsp70-1/4* double mutant

**Fig. 5:**
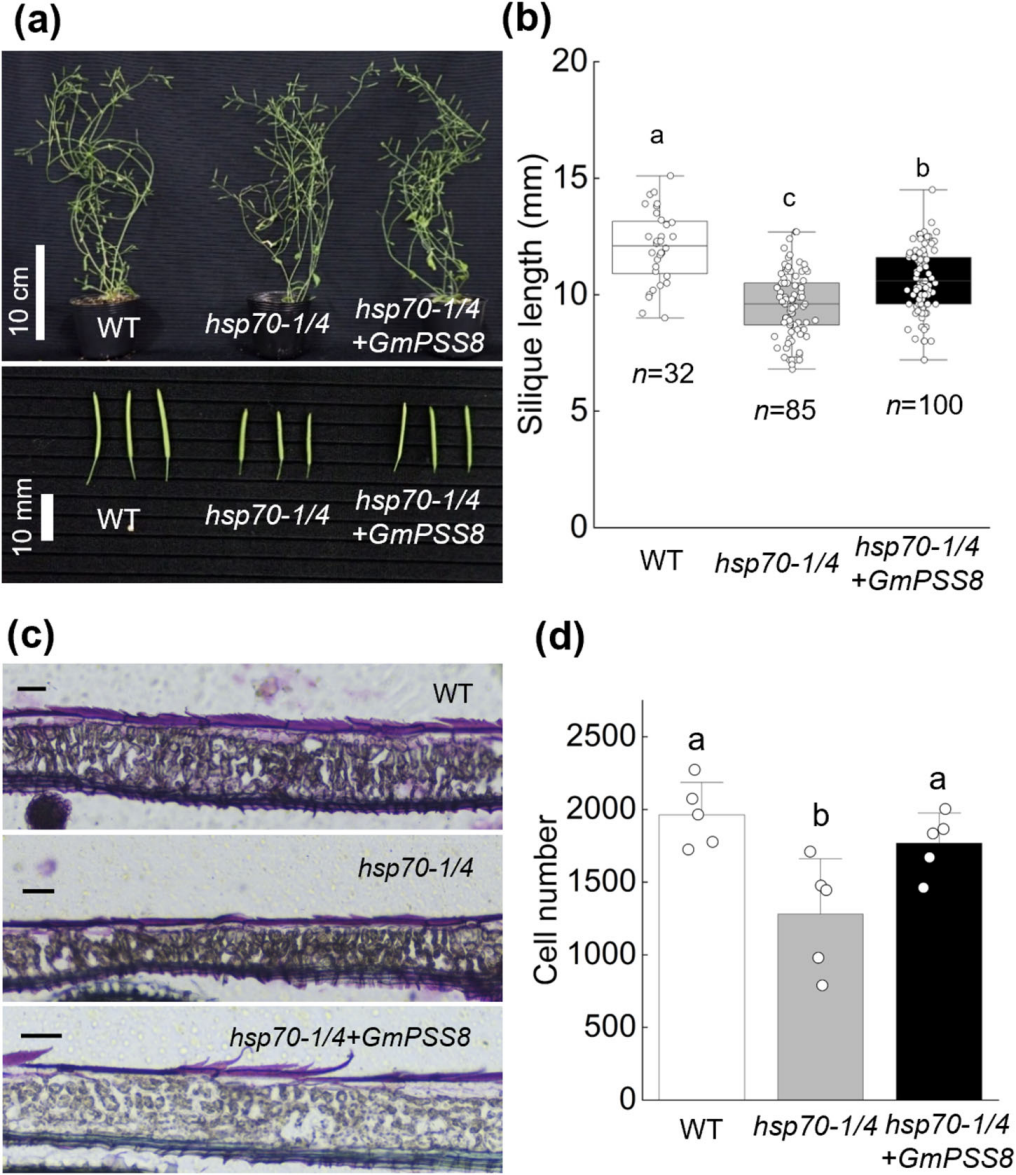
The effect of over-expression of *GmPSS8* on *Arabidopsis hsp70-1/4* double mutant. **a** The plant phenotypes and silique images of 6-week-old col-0, *hsp70-1/4* double mutant and complemental lines. Col-0 was the wild-type control. **b** The length of fully expanded siliques (One way ANOVA, *P* < 0.05). **c** Cross section images of silique wall; scale bar = 50 μm. **d** Cell number of fully expanded silique wall. (One way ANOVA, *P* < 0.05, *n* = 5). Error bars indicate SD.

We introduced *GmPSS8* into *Arabidopsis hsp70-1/4* double mutants which is short siliques^34^ to analyze the function of *GmPSS8* in the fruit development. *GmPSS8* overexpression lines showed longer siliques than the double mutants (Fig. 5a). Indeed, Silique length was 12.0 mm in Col-0, 9.6 mm in *hsp70-1/4* and 10.7 mm in complemental lines (Fig. 5b). Cell number in *GmPSS8* overexpression was more than that in *hsp70-1*/*4* and was restored to Col-0 (Fig. 5c, d). Thus, these results suggested that *GmPSS8* was involved in increasing silique development in *Arabidopsis*.

## Discussion

Our results demonstrated that longer cell proliferation lead to longer pods in Tc. Pod length in Tc and Iy was the similar until 3 DAP, but that of Tc was longer from 4 DAP. Both cell number and cell size are important to organ size. Our results showed that cell division took place before cell expansion in both cultivars. As cell number and area were similar in both cultivars at 2 DAP, pod growth was similar until then. At this time, cell number reached the maximum in Iy, while it continued to increase in Tc (Fig. 1c). Consequently, cell number was greater in Tc at 4 DAP but cell area was not.

A major QTL for pod length, *qPSS*, was detected between markers Sat_227 (10.7 cM) and Satt698 (41.7 cM) on Chr. 2 in all 3 years (Fig. 2, Supplementary Table 1). Other QTLs were also detected in different years (Supplementary Table 1). A possible explanation for the difference in detection is that pod length is a complex trait determined by many factors; the pod length of the RILs had a continuous frequency distribution. Microarray and eQTL analysis detected *GmPSS8* at *qPSS* as a *cis*-eQTL (Table 1, Fig. 2c). The expression of *GmPSS8* was regulated by *Tc-specific-1*, which has a 5′-UTR Py-rich transcription enhancer in the *GmPSS8* promoter, resulting in higher transcript levels in Tc (Fig. 3). This 5′-UTR Py-rich stretch enhances gene transcription in various plant species and a virus^35–38^. The results support the positive additive effect of *qPSS* and the *cis*-eQTL of *GmPSS8* (Fig. 2).

*GmPSS8* encodes an HSP70, which belongs to an evolutionarily conserved group of proteins present throughout the prokaryotes and eukaryotes; in *Arabidopsis*, they play roles in development, plant shape, stress responses, and hormonal regulation^34^ (Table 1). Our complementation tests showed that the introduction of *GmPSS8* in *hsp70-1/4* rescued short silique phenotypes (Fig. 5). These results suggested that the function of *Arabidopsis HSP70-1* and *HSP70-4* on fruit development was conserved in *GmPSS8*.

The expression profile of *GmPSS8*, western blot of HSP70 and the results of HSP70 inhibitor treatment suggest that HSP70 participates in pod growth regulation by modifying cell proliferation (Figs. 3, 4, S4). The expression of *GmPSS8* in 10-mm pods at 0 DAP, when cell division was in progress, was the highest (Fig. 3b). Previous studies reported the high expression of HSP70 in ovary of tomato^39^ and dwarf iris^40^. As the soybean pod develops from the ovary, the high expression of *GmPSS8* suggests an important role in pod development in soybean. Furthermore, HSP70 inhibitor treatment inhibited pod growth by decreasing cell number (Fig. 4d). Pif-µ inhibits the co-chaperone and the substrate-binding activities of HSP70^41^. A recent study using pif-µ to inhibit cotton fiber development showed that pif-µ inhibited the activity of *GhHSP70-8A*, whose expression was highest at fiber initiation and declined during fiber elongation^42^. Interestingly, *GmPSS8* had high similarity with of *GhHSP70-8A* (83.4%) relative to nucleotide sequences in Tc. This similarity suggests high conservation between soybean and cotton and the similar importance of both genes in early reproductive organ growth, especially cell proliferation. The involvement of HSP70 in cell proliferation was reported in other organisms also. Yeast HSP70 actively regulates cell division^43^. In mammals, HSP70 is associated with cell proliferation control through the cell cycle cascade^44^. In rapeseed, HSP70 may be involved in embryogenesis, which relies on cell proliferation^45^. In our study, pif-µ treatment decreased both pod length (Fig. 4b), explaining the longer pod phenotype in Tc (Fig. 1) as being due to longer cell proliferation. Thus, the difference in pod length between cultivars might be due to differential cell proliferation in pod tissue under cell cycle control associated with the transcriptional regulation of *GmPSS8*.

Since fruit size is important in crop yield and domestication, fruit size genes have been investigated. Transcriptome analysis between large- and small-pod pools of rapeseed grouped numerous genes differentially expressed in the pod wall into the “misc”, “protein”, “cell wall”, “secondary metabolism”, “hormone metabolism”, and “development” categories, showing that the differential developmental and metabolic status of the pod wall between the pools is complex^46^. Among known genes for fruit size, *fw2.2* (*SlCNR*), *SlARF9*, and *fw3.2* (*SlKLUH*) in tomato^23–25^ and *BnARF18*, *miR160*, and *BnCYP78A9* in rapeseed^22,26,27^ regulate cell proliferation. No homologs of these genes corresponded to *GmPSS* genes or other QTLs for pod length (Table 1, Fig. 2a). This implies the existence of unknown pathways in soybean through which the regulation of cell proliferation is modified by *GmPSS8*.

In rape seed, it is reported that increasing pod length results in expanding pod wall surface area, leading to more production of carbohydrates in pod and seed^46^, suggesting the important role of fruit size modulation on seed production. Soybean pods also photosynthesize^3^. Shading pods result in a reduction in seed weight of 13% to 14%^47^, suggesting the importance of photosynthesis in soybean pods for seed production. Therefore, it is considered that increasing pod length result in more accumulation of photosynthates in pods. One of the other strategies for improving seed production is physical restriction of seed growth by pods. Some articles reported that soybean seed growth is restricted by pod size^8,9^. Pod length was shortened by treatment of brassinazol, an inhibitor of brassinosteroid biosynthesis, resulting in reduced seed weight than control plants^48^, which the same phenomenon is also observed in fava beans^49^. Consequently, manipulation of pod length is possible to use as a controller of seed size. Taken together, genetic alteration using *GmPSS8* or other modifications of pod length might improve seed production.

Fruit size genes, were correlated with other factors for cell proliferation^50^. For example, *Pafw2.2* gene is negatively correlated with cell division activity, opposite the pattern　of the cell division marker genes in avocado^51^. Not only cell cycle pathways, many interaction with phytohormones, transctription factors, metabolic pathways and other proteins were also involved in organ size manipulating cell proliferation^52^. Besides *GmPSS8*, it is possible that pod length is controlled by many complex factors in soybean like other fruit-baring species.

In this study, our results showed that *GmPSS8* regulated pod length in soybean, however, the involvement of other factors and the underlying mechanism in detail remained to be further elucidated, which are considered as the goal of the next study.

In conclusion, we propose that *GmPSS8*, an HSP70 member, was determined as a gene for pod length of soybean by regulating cell number during early pod development. The difference in pod length was caused by the difference in cell number between cultivars. Gene expression of *GmPSS8* is regulated by *Tc-specific-1*, which includes an enhancer motif. Inhibition of HSP70 resulted in shorter pods with decreased cell number. Overexpression of *GmPSS8* increase silique length with increasing cell number in *Arabidopsis hsp70-1/4* double mutants. This study provides information on the regulation of pod length through the control of cell number via the expression of *GmPSS8* in soybean.

## Methods

### Plant materials and growth condition

Seeds of soybean (*Glycine max* (L.) Merr.) “Tachinagaha” (Tc), “Iyodaizu” (Iy), and 91 recombinant inbred lines (F7–9, RILs) derived from “Tachinagaha” × “Iyodaizu” were grown at Kyushu University (33°37′N, 130°25′E). Tc has large pods and Iy has small pods. Three seeds per pot were sown in 1/5000-a Wagner pots supplied with 5 g/pot of compound fertilizer (N:P:K, 3:10:10) and 5 g pot^−1^ of magnesium lime. Plants were thinned to one per pot after the emergence of the first trifoliate leaf. Plants were grown in 2013, 2014 and 2015.

For expression, QTL and microarray analysis, 2 pods per plant of Tc, Iy and 91 RILs were collected at 6 DAP (pods were 10 mm at 0 DAP), frozen immediately in liquid nitrogen, and then stored at –80 °C.

For gene expression analysis in various tissues, flower, leaf, stem, petiole and root samples were sampled from three plants per cultivar. Pods at each growth point, 0 (10-mm pods), 2, 4, 6, 8 and 10 DAP, were randomly sampled from at least 10 plants per cultivar.

### Measurement of pod length

The length of full-size pods was measured at the R5.5 (>5 mm seed) to R6 (full seed) stage. We measured pod length in ImageJ software as the distance from the pedicel to the apex in digital images taken with a high-resolution scanner or a digital camera.

### Measurements of cell area and cell number in pod tissue

Pods were fixed and stored in FAA (ethanol:acetic acid:formaldehyde = 45:5:5 v/v/v). Before cutting, samples were substituted with 30% sucrose and embedded in optimal cutting temperature compound. Pods at 0 DAP and 2–10 DAP were sectioned longitudinally at 2 µm and 8 µm thick, respectively, on a cryostat microtome (CM1905, Leica). The sections were stained with 0.05% toluidine blue and observed under a light microscope (BZ-X710, Keyence). Cell area and cell number were determined in cell-count software (BZ-H3C, Keyence).

### RNA extraction and gene expression analysis

RNA was extracted from various tissues by using the SDS/phenol/LiCl method. cDNA was synthesized from 1 µg RNA with ReverTra Ace reverse transcriptase (Toyobo) according to the manufacturer’s procedures. Real-time PCR was performed using 1 µL of cDNA as a template with 10 µL of SYBR Green Realtime PCR Master Mix Plus (Toyobo), 0.1 µL each of 100 µM forward and reverse primers (Supplementary Table 4), and 8.8 µL of sterilized water in a real-time PCR amplifier (MJ Mini, Bio-Rad). PCR conditions were 95 °C preincubation for 2 min, followed by up to 40 cycles of 95 °C for 20 s, a primer-dependent temperature for 20 s, and 72 °C for 30 s. Expression was calculated relative to that of *GmEF1b* (*Glyma13g04050*).

### Microarray analysis and data processing

Microarray analyses were performed using custom soybean 60-mer oligo microarray chips (Agilent Technologies) containing 42,034 oligonucleotides based on soybean full-length cDNA sequences. Microarray data are deposited in the NCBI Gene Expression Omnibus (GEO; https://0-www-ncbi-nlm-nih-gov.brum.beds.ac.uk/geo/) with accession number GSE136772. *GmPSS8* in our article is referred to GmPSS9 in the chip data (GSE136772). Both genes are annotated as “*Glyma02g10320*”.

### QTL (eQTL) detection in RILs

Using 234 SSR markers of *G. max* and RILs^53^ we constructed a genetic linkage map consisting of 20 chromosomes spanning 2653.2 cM with an average distance of 11.3 cM between markers. QTL (eQTL) analysis was performed by composite interval mapping in QTL Cartographer v. 2.5 software (http://statgen.ncsu.edu/qtlcart/WQTLCart.htm). The map was scanned at a 1-cM resolution and the LOD threshold for each trait was calculated using 1000 permutations, corresponding to *P* < 0.05. Pod length of RILs were used for QTL mapping, and relative expression of *GmPSS1, 2, 8,* and *13* were used for eQTL analysis.

### Screening methods for *GmPSS*s

The genomic position of *qPSS* (between Sat_227 and Satt698) was identified using the soybean genetic map in Soybase (https://www.soybase.org/). Genes significantly upregulated in Tc or Iy was annotated the Glymal gene call (ID format = glymal #g #####) corresponding to each gene on microarray data. Among these genes, we picked up 18 genes including *qPSS* based on the soybean genetic map in Soybase.

### Analysis of *GmPSS8* promoter and coding sequences

Fragments of the *GmPSS8* promoter sequences and coding sequences (CDS) from Tc and Iy were amplified from genomic DNA and were subcloned into the *pTAC-2* vector using a DynaExpress TA PCR Cloning Kit (BioDynamics Laboratory Inc.). To obtain fragments of *GmPSS8* coding sequence (CDS) specifically, CDS fragment including 5’ or 3’ untranslated region (UTR) were amplified from cDNA synthesized from pod in Tc and were inserted into the *pTAC-2* vector, designated as *pTAC-2-GmPSS8 5’-3’UTR*.

The promoter and CDS fragments were sequenced with primers listed in Supplementary Table 4. The PlantCARE database of known plant cis-acting elements (http://bioinformatics.psb.ugent.be/webtools/plantcare.html) was used to search for cis-acting regulatory elements in the sequenced promoter.

### Subcellular localization of GmPSS8 protein

To obtain *35S::sGFP(S65T)-GmPSS8CDS*, *GmPSS8*CDS fragments with *BglII* on 5’and *XhoI* site on 3’ side were amplified from *pTAC-2-GmPSS8 5’-3’UTR* with cloning primers (Supplementary Table 4), and digested with *BglII* and *XhoI* after that. *35* *S::sGFP(S65T)* control vector was double-digested with *BglII* and *SalI*. The resulting products were ligated using Ligation-Convenience Kit (Nippon Gene Co., Ltd.).

3*5S::sGFP(S65T)-GmPSS8CDS* or *35S::sGFP(S65T)* control vector were delivered with gold particles to onion epidermal cell placed on MS medium using Biolistic PDS-1000/He particle Delivery system (Bio-Rad). After bombardment, the onion epidermis was incubated at 20 °C on 1/2 MS plate under dark overnight, and then the GFP fluorescence was observed under a light and fluorescence microscope (BZ-X710, Keyence).

### Protein accumulation of HSP70

Frozen pod tissues were homogenized in liquid nitrogen and diluted 2 mL of lysis buffer solution (1×TBS (25 mM Tris-HCl (pH 7.4), 137 mM NaCl), 5 mM EDTA, 10%(v/v) glycerol, 0.5%(v/v) Triton-X 100, 0.05% β-mercapto ethanol, 1×protease inhibitor cocktail 0.1 mM PMSF). Homogenates were centrifuged at 13,000 rpm at 4 °C for 20 min, the supernatants were transferred as total protein extracts, and total protein was quantitated by Bradford assay.

One µg of total protein was boiled with SDS sample buffer (125 mM Tris-HCl (pH 6.8), 1%(v/v) β-mercapto ethanol, 2% (w/v) SDS, 20%(v/v) glycerol, 0.08% (w/v) BPB) for 10 min at 65°C. Each protein sample was loaded onto a 12.5% SDS-polyacrylamid gel and separated at a constant 20 mA for 150 min running buffer (25 mM Tris base, 192 mM glycine, 0.1% (w/v) SDS). Proteins were stained in CBB staining buffer (0.25% CBB-R250, 40% (v/v) methanol, 10% (v/v) acetic acid) Proteins were transferred at a constant 100 V for 90 min. onto a 0.45 µm PVDF membrane (Merck Millipore) in ice-cold transfer buffer (25% (v/v) methanol, 25 mM Tris, and 192 mM glycine). The transferred membrane was washed in 99% methanol and TBS-T (1×TBS, 0.1% (v/v) Tween-20), then blocked in 3% skim milk powder in TBS-T for 1 h at a room temperature and washed with TBS-T. The membrane was incubated in primary antibody solution (0.01% (v/v) anti-cytoplasmic HSP70 antibody (Agrisera #AS08 371) in blocking buffer) and washed with TBS-T. The membrane was incubated in secondary antibody solution (0.05% (v/v) anti-rabbit IgG-horseradish peroxidase (HRP) in blocking buffer) and washed with TBS-T.

After membrane washing antibody-reactive protein bands were visualized with Piece ECL Plus Western Blotting Substrate (Thermo Scientific) according to the manufacture’s procedure and observed using Chemiluminescent Imaging System (WSE-6100, ATTO). HSP70 band intensities were obtained with CS Analyzer4 (ATTO).

Uncropped gel and membrane images are provided in Supplementary Fig. 9.

### Reporter assay

To generate the *35S::Renilla luciferase (hRLUC)* reporter and *35S::Firefly luciferase (fLUC)* control vectors, *hRLUC* and *fLUC* amplified from the *pGL4* vector series were inserted into the *BamHI/SacI*-digested *pBI221* vector, where they were ligated with the use of NEBuilder HiFi DNA Assembly Master Mix (New England Biolabs Japan Inc.). To construct other reporter vectors (−792 Tc, −1472 Tc, −1552 Tc, −2186 Tc, and −2186 Iy fused with *hRLUC*), we double-digested the *pBI221* vector containing hRLUC with NcoI/XbaI. The corresponding *GmPSS8* promoter fragments were amplified from *pTAC-2-GmPSS9pro*. as a template with primers listed in Supplementary Table 4 and inserted into the *NcoI/XbaI* sites of *pBI221*.

DNA-coated particles with the same concentrations of reporter and internal control vectors were delivered to 2-cm × 2-cm leaf discs of soybean (“Williams 82’), which has an Iy-type polymorphism lacking *Tc-specific-1* and *-2*, on MS medium, by using a Biolistic PDS-1000/He Particle Delivery system (Bio-Rad). The treated leaf discs were incubated at 22 °C on the MS medium under 24-h light for 2 days. Luciferase activity in the leaf discs was measured with a Dual-Luciferase Reporter Assay System (Promega) on a Luminescencer PSN AB-2200 (Atto Co.) according to the manufacturers’ instructions.

### HSP70 inhibitor treatment

Just before use, the HSP70 inhibitor pif-µ was dissolved in dimethyl sulfoxide (DMSO) and diluted to 100 µM with 0.05% DMSO and 0.05 A% Triton X-100. The control contained only DMSO and Triton-X 100.

To observe the effect of HSP70 inhibition on plant height and pod growth, we grew Tc plants at 25 °C under ambient light. Plants were sprayed once a day for 10 days after the development of the first trifoliate leaf. Plant height was measured at 10th day. For measurement of pod growth, pods >5 mm were treated once a day for 12 days with a paint brush. Pod length was measured at 12th day, and pods were picked and soaked in FAA (ethanol:acetic acid:formaldehyde = 45:5:5 v/v/v) to measure cell number and area.

### Complementation tests of *GmPSS8* in *Arabidopsis hsp70-1/4* mutant

To obtain *35S::GmPSS8CDS* vector for overexpression, designated as *pGWB502-GmPSS8CDS*, full length of *GmPSS8CDS* fragment was cloned from with cloning primers (Supplementary Table 4) from *pTAC-2-GmPSS8CDS*. *pGWB502* empty vector was double-digested with *XbaI* and *SalI*. The amplified *GmPSS8CDS* was inserted into digested *pGWB502* vector using NEBuilder HiFi DNA Assembly Master Mix (New England Biolabs Japan Inc.).

Arabidopsis seeds, seedlings and plants were grown in a controlled environment cabinet at 22 °C in 16/8-h light/dark regime. *Arabidopsis* seeds were germinated on an MS medium. Two-week-seedlings were transplanted to soil.

Two *Arabidopsis* T-DNA insertion mutants, *hsp70-1* (SALK_135531) and *hsp70-4* (SALK_088253) were used to generate *hsp70-1/4* double mutant by crossing, F1 and F2 generations were genotyped with specific primers (Supplementary Table 4). An Agrobacterium strain EHA105 containing *pGWB502-GmPSS8CDS* was infected to immature bud and unopened flower of *hsp70-1/4*.

Col-0 (as a control line), *hsp70-1/4* double mutants and T_3_ plants (complemental lines) were used for morphological analysis. Silique length was obtained by measuring green mature silique. To measure cell number in silique tissues, full-expanded siliques collected were fixed and stored in FAA (ethanol:acetic acid:formaldehyde = 45:5:5 v/v/v). Before cutting, samples were substituted with 10% sucrose and embedded in optimal cutting temperature compound. Mature siliques were sectioned longitudinally on a cryostat microtome (CM1905, Leica). The sections were stained with 0.05% toluidine blue and observed under a light microscope (BZ-X710, Keyence). Cell number was determined in cell-count software (BZ-H3C, Keyence).

### Statistics and reproducibility

Statistical analyses were performed in SPSS software version 28.0.0.0 (IBM). Differences among treatments were analyzed by one-tailed student’s *t* test and Tukey’s test.

For growth parameters comparisons, 11–13 pods (Fig. 1b), five plants (Fig. 4a), 19 pods (Fig. 4b), 32–100 siliques (Fig. 5) or 44–55 siliques (Supplementary Fig. 8b) pods or siliques were used as biological replicates. For measurement of cell number and cell area of pods or siliques, five pods or siliques were used (Figs. 1d, 4d, 5d, Supplementary Fig. 8d). The data for QTL and eQTL analysis (Fig. 2, Supplementary Fig. 1, Supplementary Table 1, 2 and 3) was obtained from at least three plants per line among 91 RILs. QTL analysis of pod length was performed for three years (2013. 2014 and 2015), and eQTL analysis was done only 2015. For gene expression analysis and reporter assay, three to five pods or other organs were used (Fig. 3a, b, d, Supplementary Fig. 2, 5). Western blotting used three pods as biological replicates. Statistical methods,　sample sizes and number of replicates are stated for each figure captions.

### Reporting summary

Further information on research design is available in the Nature Portfolio Reporting Summary linked to this article.

### Supplementary information

Supplementary Information

Description of Additional Supplementary Files

Supplementary Data 1

Supplementary Data 2

Reporting Summary

## Data Availability

Microarray data is available at NCBI GEO with accession number GSE136772. A gene list of the up-regulated genes in Tc and Iy for Fig. 2b was provided as Supplementary Data 1. Almost all source data underlying the graphs presented in the main and Supplementary Figs. exception for Fig. 2b is provided in Supplementary Data 2. The other data supporting the findings of this study are available from the corresponding author upon request.
