# Supplementary material for: Heat shock protein 70 is associated with duration of cell proliferation in early pod development of soybean
Source: Commun Biol. 2024 Jun 21;7:755. doi: 10.1038/s42003-024-06443-8 (PMC11192946; doi:10.1038/s42003-024-06443-8)
Supplement: Supplementary file 1 — Supplementary Information [file 42003_2024_6443_MOESM1_ESM.pdf]

Supplementary Table 1 QTLs for pod length detected above LOD threshold by CIM in 3 years.

Year	Chr.	Marker Interbal / Position (cM)	LOD	R^2 (%)	A.E. (mm)
2013	2	Satt216-Satt698 / 16.4-41.5	3.3	11.38	0.914
2014	2	Sat_227-Satt216 / 10.7-16.4	3.2	9.49	0.708
	3	Satt521-Sat_306 / 66.1-88.9	4.4	14.42	0.938
2015	2	Sat_227-Satt216 / 10.7-16.4	4	11.96	0.995
	12	Satt676-Sat_158 / 106.3-119.0	3.5	10.27	0.921

Chr., chromosome number; LOD, logarithm of odds; R^2, percentage of variance explained; A.E., additive effect.

LOD thresholds were estimated by permutation test (1000 replications, $P < 0.05$).

Supplementary Table 2 The eQTL for target genes *GmPSS1, 2, 8* and *13*.

	Chr.	Marker interval	Position (cM)	LOD	R^2 (%)	A. E.
GmPSS1	11	Sat_095-Satt415	93.8-98.4	3.1	10.57	0.0025348
	12	Satt052-Satt676	95.7-106.3	4.1	14.72	0.0029961
GmPSS2	16	Sat_339-Satt011	22.6-83.6	11.5	34.8	0.0041673
GmPSS8	2	Satt698-Satt634	41.5-60.6	4.7	16.58	0.1686773
GmPSS13	12	Satt302-Satt637	125.3-128.3	2.8	10.38	-0.0011546

Chr., chromosome number; LOD, logarithm of odds; R^2, percentage of variance explained; A.E., additive effect.

LOD thresholds were estimated by permutation test (1000 replications, $P < 0.05$).

Supplementary Table 3 List of cis-acting elements in *T-specific-1* and *-2* obtained from PlantCARE

Motif name	sequences	Motif functions
T-specific1		
5'UTR Py-rich stretch	TTTCTCTCTCTCTC	cis-acting element conferring high transcription levels
ABRE	TACGTG	cis-acting element involved in the abscisic acid responsiveness
Box_4	ATTAAT	part of a conserved DNA module involved in light responsiveness
Box_1	TTTCAAA	light responsive element
CAAT Box	CAATT etc.	common cis-acting element in promoter and enhancer regions
CCAAT Box	CAACGG	MYBHv1 binding site
G-Box	CACGTA etc.	cis-acting regulatory element involved in light responsiveness
GAG-motif	GGAGATG	part of a light responsive element
GCN4_motif	CAAGCCA	cis-regulatory element involved in endosperm expression
MNF1	GTGCCC(A/T)(A/T)	light responsive element
MRE	AACCTAA	MYB binding site involved in light responsiveness
TATA Box	TAATA	core promoter element around -30 of transcription start
TCA-element	CAGAAAGGA	cis-acting element involved in salicylic acid responsiveness
TGA-element	AACGAC	auxin-responsive element
Circadian	CAANNNNATC	cis-acting regulatory element involved in circadian control
T-specific2		
AAGAA-motif	GAAAGAA	
HSE	AGAAAATTCG	cis-acting element involved in heat stress responsiveness

Supplementary Table 4 Primers used in this study.

**Real-time PCR**	Primer sequences (5' to 3')
GmPSS1 F	AAGTGGCTCCCTGCAAGTAGGA
GmPSS1 R	GCAGTGGCTGGAACTGATTGTGG
GmPSS2 F	GGTGAGTGATGTCAGGGATGCCAA
GmPSS2 R	CGTCGTTGGGCAACAAGTCTGT
GmPSS3 F	TCACAAGATCCCAAACCGCCTCC
GmPSS3 R	CACCAGCGCACACCGATTATCCT
GmPSS4 F	ATCCCCACTCCAGAGCCAAGGTA
GmPSS4 R	TGAAGACCGAGCCTGCTCCCTTA
GmPSS5 F	AGAGGACCCCATCAGAGGAATGGT
GmPSS5 R	ATTGTTACGCACCTCAATGGGCCG
GmPSS6 F	GCATTTGCCTCCTCTTCTCAAGTCT
GmPSS6 R	CTGCAGCTGATGCTGCCATGA
GmPSS7 F	GCAGCATTGTCCCAAGGCCA
GmPSS7 R	TGTGCTGATTGGAAGTGCGGGT
GmPSS8 F	GATGAAGGAGCTTGAGAGCAC
GmPSS8 R	ATCCACCACAAACCCATCTC
GmPSS9 F	CCTCATCACTCTCTCCAACGCCA
GmPSS9 R	GCGATTCTCGCGTTTCGCTTGTT
GmPSS10 F	CGCACGGTTCTTCCTCAGTGC
GmPSS10 R	TGCCTGTCTTGCCGCTGCTT
GmPSS11 F	GCTATGCTACCAAGCGCACAACG
GmPSS11 R	AGGAAAACGCCACTGGCACATCT
GmPSS12 F	TGGGCTTGGGGACAAGAAGCA
GmPSS12 R	CCACGGCAGCAACACGATCT
GmPSS13 F	GCTCCCTTGTGCCTCTGTCACT
GmPSS13 R	TCGAAGCCGCCAATCGAGGT
GmPSS14 F	TAGCCCAGCGGCTTCTCAACATC
GmPSS14 R	CAGGTTTTGTCGGGTTCGGGGAA
GmPSS15 F	TCTTGGTGTTCTTCTCCGCCGTG
GmPSS15 R	ACCTCGATCGGGTTAGAACGCAC
GmPSS16 F	TGATGGATGAGAAAGAGTGGCAGGT
GmPSS16 R	TGGGTTTCTACGCCTTTCCACGG
GmPSS17 F	AAAGAGCTACCTGAGAAGGCGGC
GmPSS17 R	ACCCATCTAAGCCTTGGAATTGCTC
GmPSS18 F	CCGCCGATTCACGGTTGTTCAC
GmPSS18 R	CCTGACAACCAATCAAACACTGCGG
GmEF1b F	GAAGACAAAAAGGCAGCAGAGGAA
GmEF1b R	TCCACAGATACAAGGTCATCGACA
**Sequense analysis**	
GmHSP70-CDS F	ATGAAGCTATGGCCATTTAAGGT
GmHSP70-CDS R	TTAGTCAACTTCCTCGATCTTGGG
GmHSP70-5UTR F	ACGCCACTCGTATTACACATT
GmHSP70-3UTR R	TCCACCACAAACCCATCTCAT
GmHSP70-pro F	GGACGTAACAACATCGTCCTC
GmHSP70-pro R	TATCACTCTGAACGGAGGCA
GmHSP70-337 R	ACACGTTCTTCTCACCCACG
GmHSP70-519 F	CCCTAGGGCTCTTAGGAGGT
GmHSP70-1110	GTTGTCCCTGGTTCTAGCTCT
GmHSP70-1330 F	GCTGAGGATGAGGAGCACAA
GmHSP70--1432	CAGGTCACGTGTCATCCCTC
GmHSP70--658	TACCGAAGACGGTGTTGACG
GmHSP70--1303	TTCGACGATGACAAGACCGG
**Promoter cloning and vector construction**	
Reporter PSS8proTc-792F	CCACTGAATCAAAGGCCATGGTCGAAATCATCGCCAATG
Reporter PSS8proTc-1472F	CCACTGAATCAAAGGCCATGAAAGCTAAAGAGCATTTTTGCC
Reporter PSS8proTc-1552F	CCACTGAATCAAAGGCCATGCGCGGAAGAAATGTTCCAC
Reporter PSS8proTc-2186F	CCACTGAATCAAAGGCCATGAACATTTCCTCTTGAGGGATG
Reporter PSS8pro3'R	GAAGCCATGGATCCTCTAGA+B39:B60ATCACTCTGAACGGAGGC
**Amplification of GmPSS8CDS**	
GmPSS8cdsF 502 XbaI	TCATTTGGAGAGAACACGGGGGACTATGAAGCTATGGCCATTTAAG
GmPSS8cdsR 502 SacI	TTGAACGATCGGGGAAATTCGAGCTTTAGTCAACTTCCTCGATC
**Genotyping for Arabidopsis T-DNA mutants**	
Hsp70-1-F	AAGGAGAAGGACCAGCTATCG
Hsp70-1-R	TCTTCGCTCTCTCACAGGAAG
Hsp70-4-F	CCAAATACGAAGCCACTTGAG
Hsp70-4-R	TACCGAAGACGGTGTTGGTAG
SALKLB1.3	ATTTTGCCGATTTCGGAAC

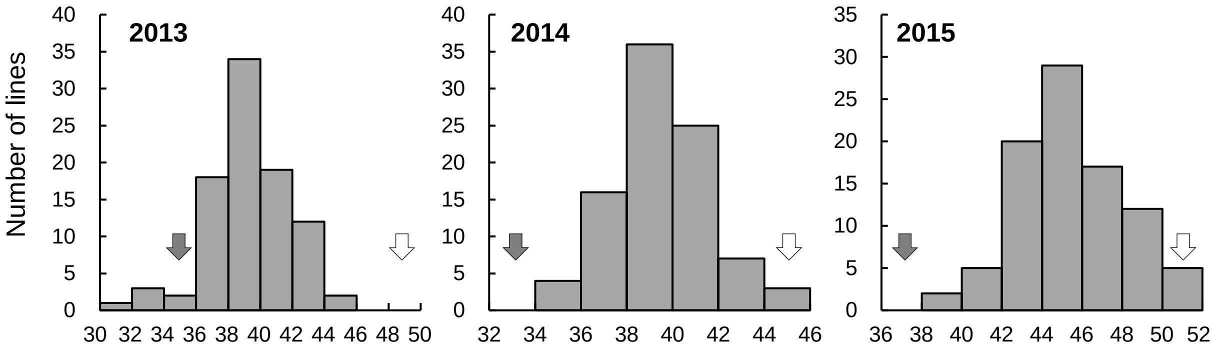

Supplementary Figure 1 Frequency distribution of pod length in 91 RILs. Arrows: white, Tc; gray, Iy.

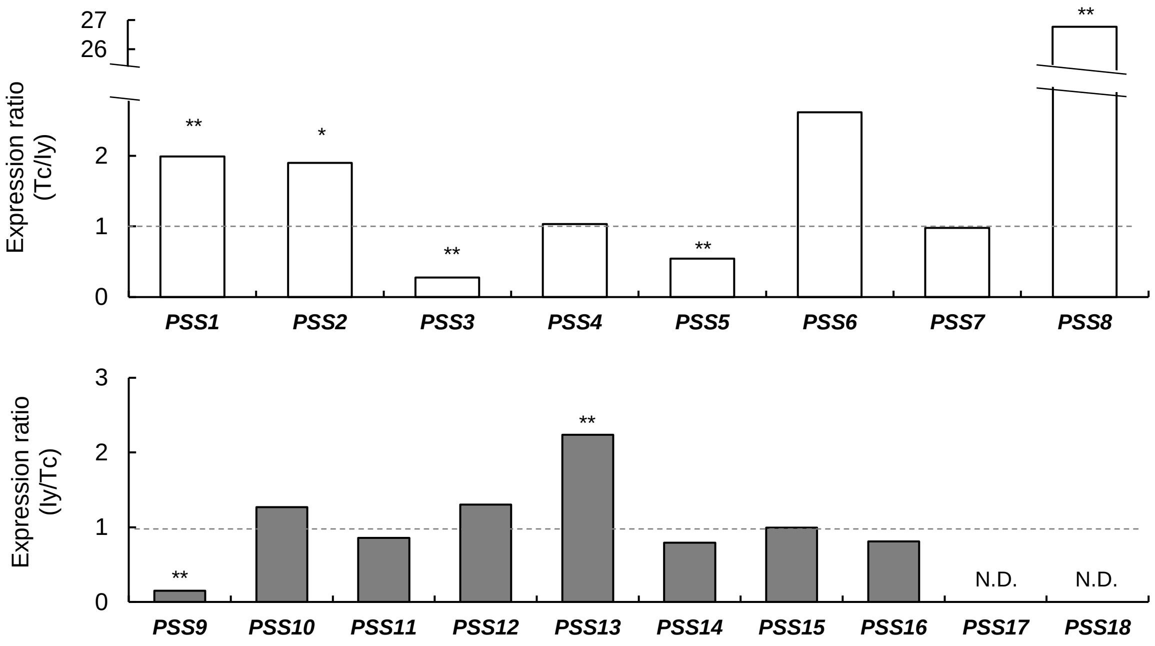

Supplementary Figure 2 Expression ratio of *GmPSSs* in developing pods. N.D., “not detected”; *n* = 3. Significance by Student’s t-test between Tc and Iy: $*P < 0.05$, $**P < 0.01$.

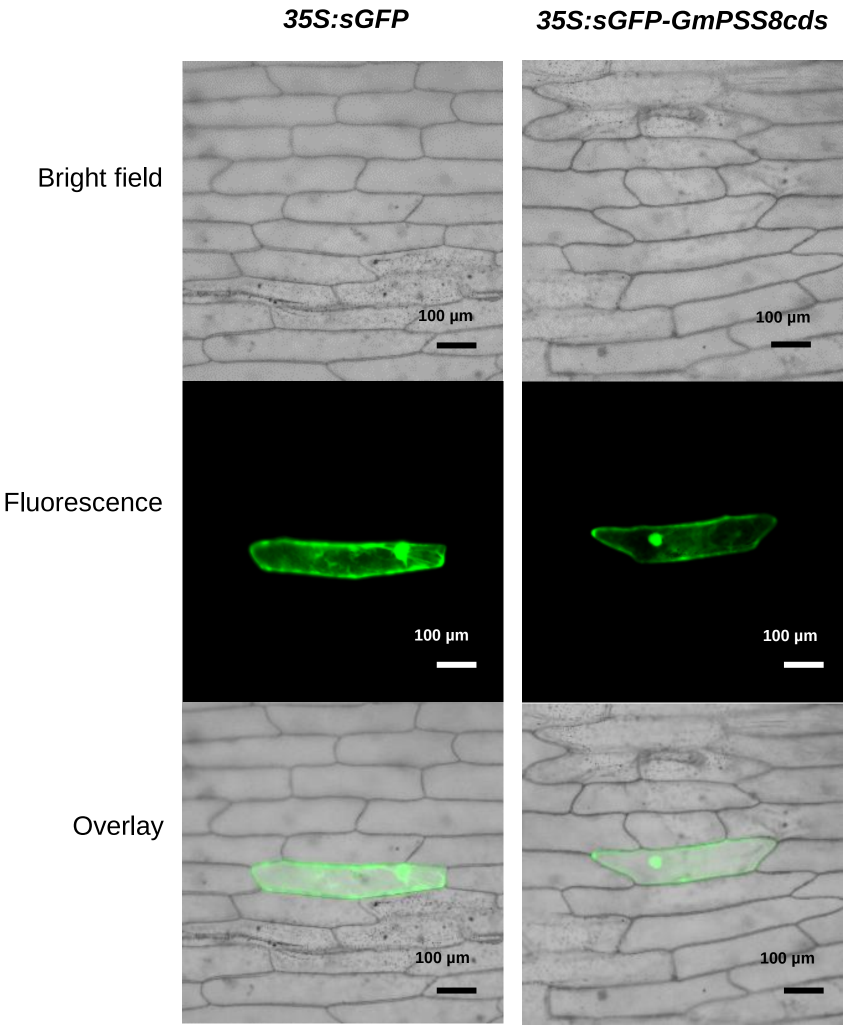

Supplementary Figure 3 Subcellular localization of *GmPSS8*.

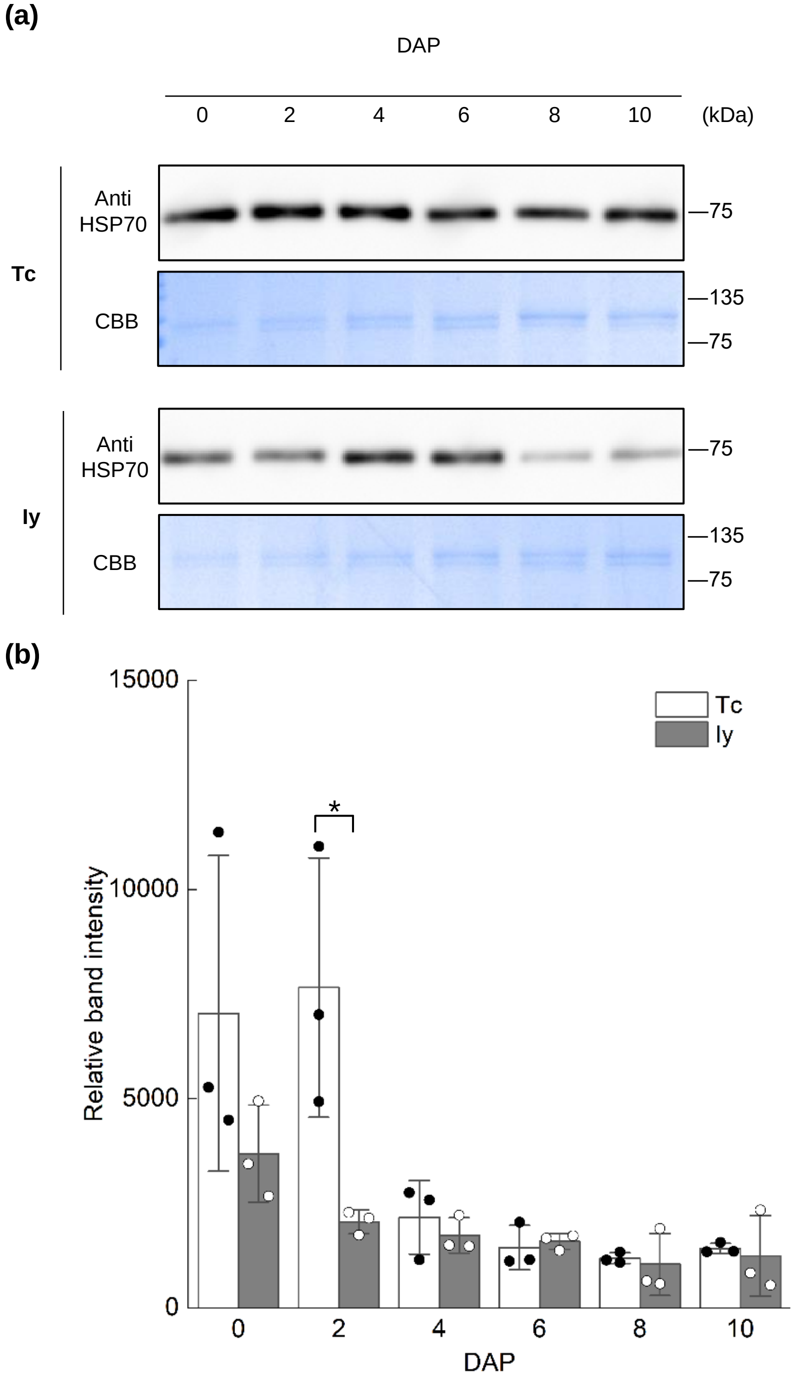

Supplementary Figure 4 Temporal accumulation of HSP70 in Tc and Iy pod (a) Immuno-blots of cytoplasmic HSP70 in developing pods in Tc and Iy. CBB was used as a control; *n* = 3. (a) Relative band intensity of Anti-cytoplasmic HSP70 against CBB staining. *n*=3. Significance by Student's t-test: *$P < 0.05$.

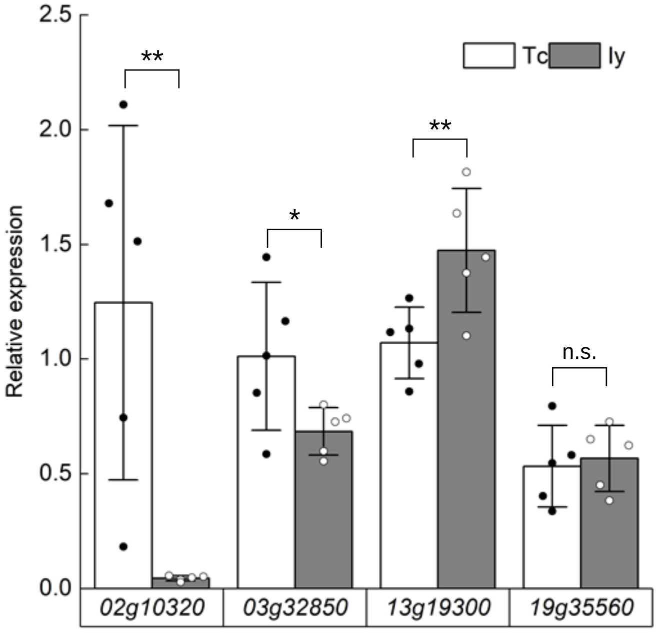

Supplementary Figure 5 Expression of *GmPSS8* (*Glyma02g10320*) and the top three highly expressed *HSP70* homologues (*Glyma03g32850*, *Glyma13g19300*, *Glyma19g35560*). Significance by Student's t-test: *$P < 0.05$, **$P < 0.01$, n.s. no significance.

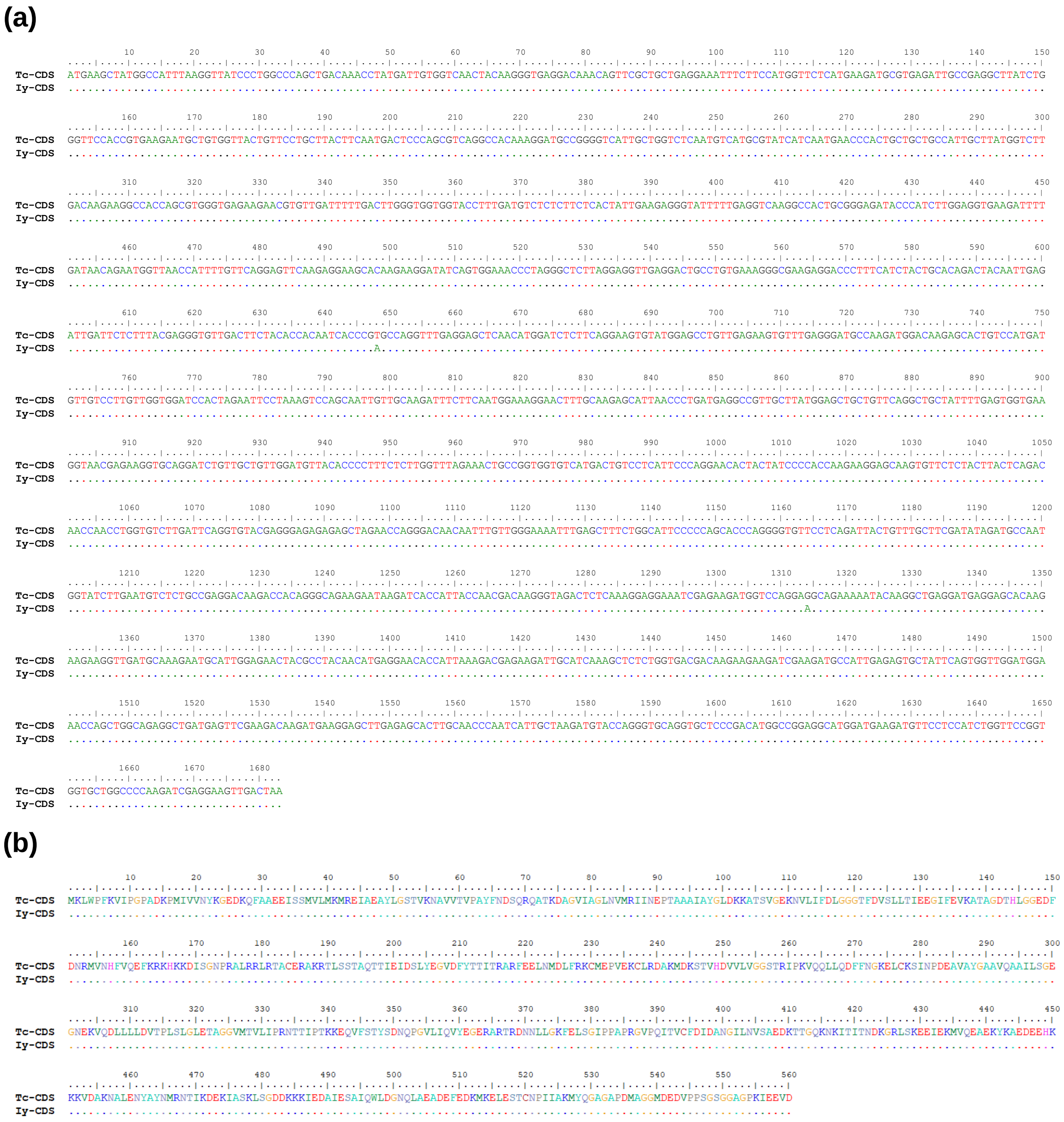

Supplementary Figure 6 Alignment of *GmPSS8* coding sequences (CDS) between Tc and Iy. (a) Nucleotide and (b) putative amino acid sequences of *GmPSS8*. Each dots(·) indicates a same base or amino acid referred to the sequences of Tc.

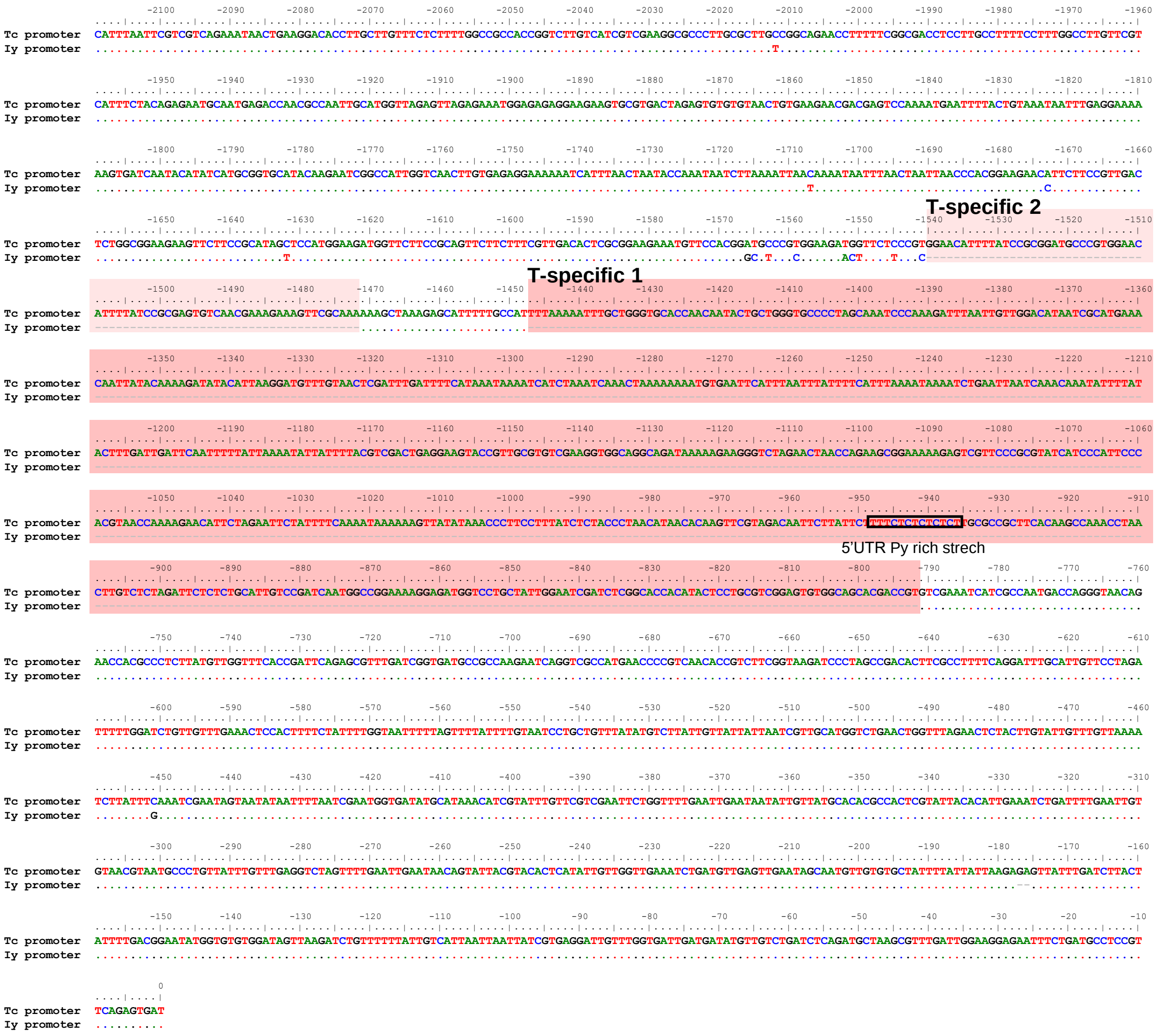

Supplementary Figure 7 Alignment of *GmPSS8* promoter between Tc and Iy. Dots (•) in Iy promoter indicate the same as in Tc. Dashes (–) indicate deletion. Box indicates 5′-UTR Py-rich stretch motif.

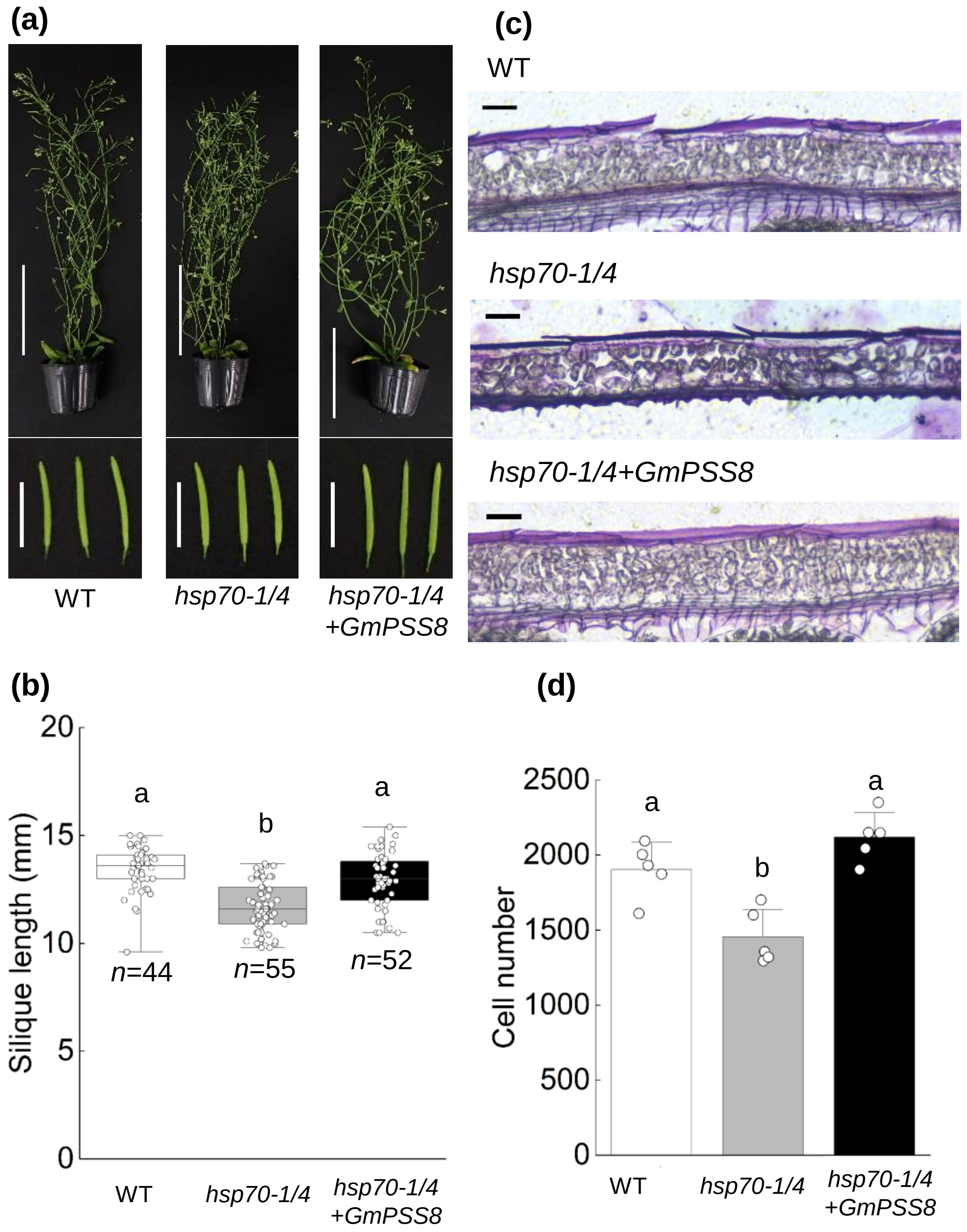

Supplementary Figure 8 The experimental replication of the effect of over-expression of *GmPSS8* on *Arabidopsis hsp70-1/4* double mutant. (a) The plant phenotypes and silique images of 6-week-old col-0, *hsp70-1/4* double mutant and complemental lines. Col-0 was the wild-type (WT) control. Scale bar = 10 cm in figures of plants; = 10 mm in figures of siliques. (b) The length of fully expanded siliques (One way ANOVA, $P < 0.05$). (c) Cross section images of silique wall; scale bar = 50 μm. (d) Cell number of fully expanded silique (One way ANOVA, $P < 0.05$, $n = 5$).

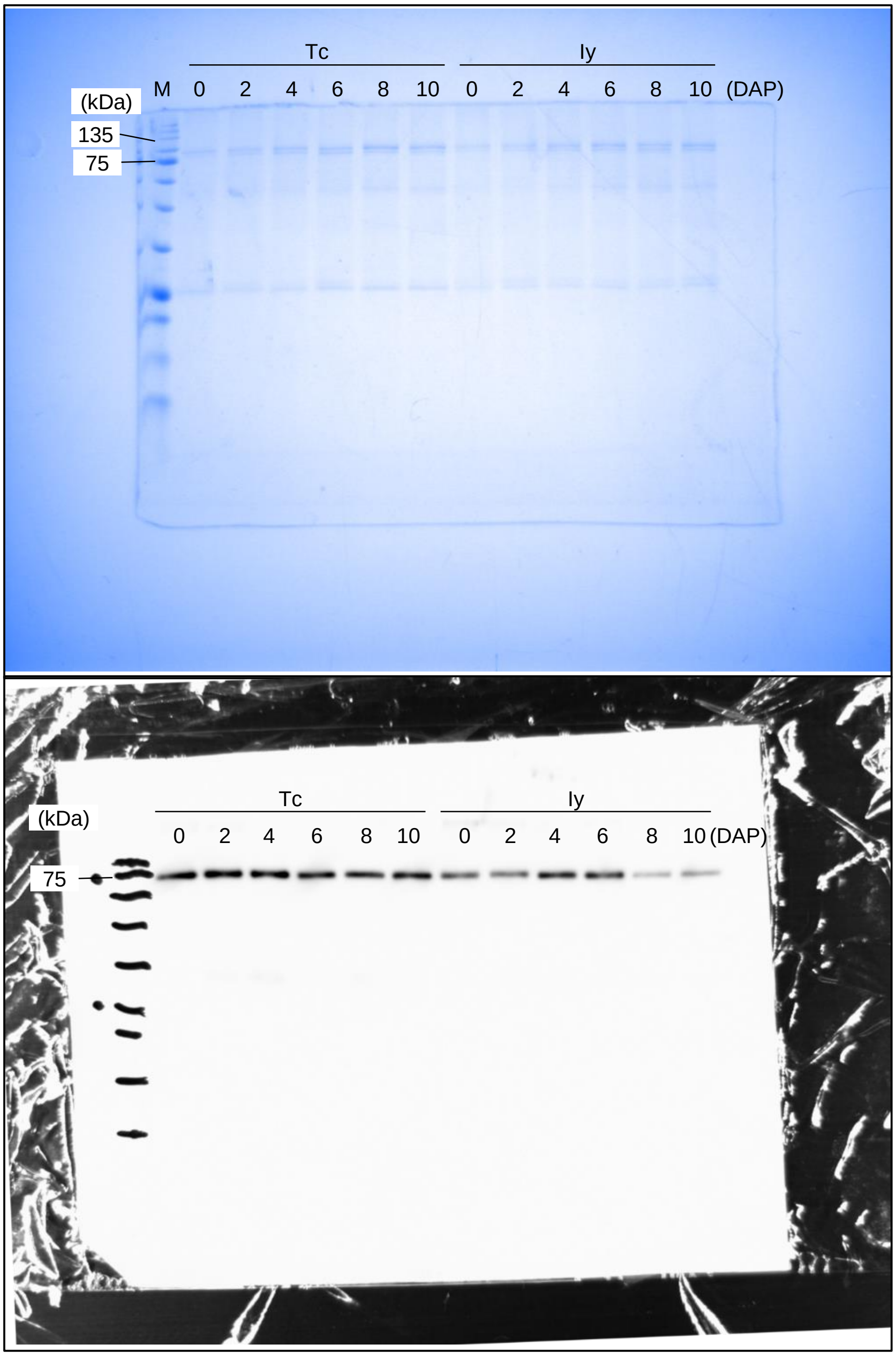

Supplementary Figure 9 Uncropped gel images in Supplymentaly figure 4
