# Supplementary material for: Heat shock protein 70 is associated with duration of cell proliferation in early pod development of soybean
Source: Commun Biol. 2024 Jun 21;7:755. doi: 10.1038/s42003-024-06443-8 (PMC11192946; doi:10.1038/s42003-024-06443-8)
Supplement: Supplementary file 2 — Description of Additional Supplementary Files [file 42003_2024_6443_MOESM2_ESM.pdf]

Description of Additional Supplementary Files

File name: Supplemental Data 1

Description: Genes differentially expressed between ‘Tachinagaha’ and ‘Iyodaizu’.

File name: Supplemental Data 2

Description: All source data underlying the graphs presented in the main and supplementary figures.
