# Supplementary material for: Heat shock protein 70 is associated with duration of cell proliferation in early pod development of soybean
Source: Commun Biol. 2024 Jun 21;7:755. doi: 10.1038/s42003-024-06443-8 (PMC11192946; doi:10.1038/s42003-024-06443-8)
Supplement: Supplementary file 5 — Reporting Summary [file 42003_2024_6443_MOESM5_ESM.pdf]

nature portfolio

Corresponding author(s): Yushi Ishibashi

Last updated by author(s): May 10, 2024

Reporting Summary

Nature Portfolio wishes to improve the reproducibility of the work that we publish. This form provides structure for consistency and transparency in reporting. For further information on Nature Portfolio policies, see our Editorial Policies and the Editorial Policy Checklist.

Statistics

For all statistical analyses, confirm that the following items are present in the figure legend, table legend, main text, or Methods section.

n/a	Confirmed	
☐	☒	The exact sample size (*n*) for each experimental group/condition, given as a discrete number and unit of measurement
☐	☒	A statement on whether measurements were taken from distinct samples or whether the same sample was measured repeatedly
☐	☒	The statistical test(s) used AND whether they are one- or two-sided<br>*Only common tests should be described solely by name; describe more complex techniques in the Methods section.*
☒	☐	A description of all covariates tested
☒	☐	A description of any assumptions or corrections, such as tests of normality and adjustment for multiple comparisons
☐	☒	A full description of the statistical parameters including central tendency (e.g. means) or other basic estimates (e.g. regression coefficient) AND variation (e.g. standard deviation) or associated estimates of uncertainty (e.g. confidence intervals)
☐	☒	For null hypothesis testing, the test statistic (e.g. *F*, *t*, *r*) with confidence intervals, effect sizes, degrees of freedom and *P* value noted<br>*Give P values as exact values whenever suitable.*
☒	☐	For Bayesian analysis, information on the choice of priors and Markov chain Monte Carlo settings
☒	☐	For hierarchical and complex designs, identification of the appropriate level for tests and full reporting of outcomes
☒	☐	Estimates of effect sizes (e.g. Cohen's *d*, Pearson's *r*), indicating how they were calculated

Our web collection on statistics for biologists contains articles on many of the points above.

Software and code

Policy information about availability of computer code

Data collection	No software was used for data collections
Data analysis	Images of soybean pods and Arabidopsis siliques and fluorescent images of onion epidermis were obtained with light and fluorescence microscope (BZ-X710, Keyence)<br>Cell number and cell area were determined in cell-count software (BZ-H3C, Keyence)<br>Pod length (soybean) and silique length were measured with ImageJ software in digital images taken with a high-resolution scanner or a digital camera.<br>QTL and eQTL analysis were conducted with QTL Cartographer v. 2.5 software.<br>Gel images and figures of western blotting were obtained with Chemiluminescent Imaging System (WSE-6100, ATTO).<br>All statistical analysis were conducted using SPSS software version 28.0.0.0 (IBM).

For manuscripts utilizing custom algorithms or software that are central to the research but not yet described in published literature, software must be made available to editors and reviewers. We strongly encourage code deposition in a community repository (e.g. GitHub). See the Nature Portfolio guidelines for submitting code & software for further information.

Data

Policy information about availability of data

All manuscripts must include a data availability statement. This statement should provide the following information, where applicable:

- Accession codes, unique identifiers, or web links for publicly available datasets
- A description of any restrictions on data availability
- For clinical datasets or third party data, please ensure that the statement adheres to our policy

All source data underlying the graphs presented in the main and supplementary figures are provided in Supplementary Data 1. The other data supporting the findings of this study are available from the corresponding author upon request.

Research involving human participants, their data, or biological material

Policy information about studies with human participants or human data. See also policy information about sex, gender (identity/presentation), and sexual orientation and race, ethnicity and racism.

Reporting on sex and gender	NA
Reporting on race, ethnicity, or other socially relevant groupings	NA
Population characteristics	NA
Recruitment	NA
Ethics oversight	NA

Note that full information on the approval of the study protocol must also be provided in the manuscript.

Field-specific reporting

Please select the one below that is the best fit for your research. If you are not sure, read the appropriate sections before making your selection.

☒ Life sciences ☐ Behavioural & social sciences ☐ Ecological, evolutionary & environmental sciences

For a reference copy of the document with all sections, see nature.com/documents/nr-reporting-summary-flat.pdf

Life sciences study design

All studies must disclose on these points even when the disclosure is negative.

Sample size	Sample size was indicated in the captions of corresponding figures.
Data exclusions	No data was excluded
Replication	All samples were obtained from at least 3 individuals.
Randomization	All samples were randomly collected.
Blinding	NA

Reporting for specific materials, systems and methods

We require information from authors about some types of materials, experimental systems and methods used in many studies. Here, indicate whether each material, system or method listed is relevant to your study. If you are not sure if a list item applies to your research, read the appropriate section before selecting a response.

Materials & experimental systems

n/a	Involved in the study	
☐	☒	Antibodies
☒	☐	Eukaryotic cell lines
☒	☐	Palaeontology and archaeology
☒	☐	Animals and other organisms
☒	☐	Clinical data
☒	☐	Dual use research of concern
☐	☒	Plants

Methods

n/a	Involved in the study	
☒	☐	ChIP-seq
☒	☐	Flow cytometry
☒	☐	MRI-based neuroimaging

Antibodies

Antibodies used	Anti-cytoplasmic HSP70 antibody (#AS08 371, Agrisera) ,anti-rabbit IgG-horseradish peroxidase.
Validation	Anti-cytoplasmic HSP70 antibody was experimentally tested within the range of recommended concentration (used in 1:10000, Agrisera)

Dual use research of concern

Policy information about dual use research of concern

Hazards

Could the accidental, deliberate or reckless misuse of agents or technologies generated in the work, or the application of information presented in the manuscript, pose a threat to:

No	Yes	
☒	☐	Public health
☒	☐	National security
☒	☐	Crops and/or livestock
☒	☐	Ecosystems
☒	☐	Any other significant area

Experiments of concern

Does the work involve any of these experiments of concern:

No	Yes	
☒	☐	Demonstrate how to render a vaccine ineffective
☒	☐	Confer resistance to therapeutically useful antibiotics or antiviral agents
☒	☐	Enhance the virulence of a pathogen or render a nonpathogen virulent
☒	☐	Increase transmissibility of a pathogen
☒	☐	Alter the host range of a pathogen
☒	☐	Enable evasion of diagnostic/detection modalities
☒	☐	Enable the weaponization of a biological agent or toxin
☒	☐	Any other potentially harmful combination of experiments and agents

Plants

Seed stocks	Seeds of soybean cultivars, "Tachinagaha", "Iyodaizu" and "Williams82", and RILs were collected in Kyushu University (33°37′N, 130° 25′E).<br>Col-0 is Arabidopsis thaliana wild-type and background of T-DNA insertion mutants.<br>Transgenic line, hsp70-1/-4+GmPSS8, was generated in this study.<br>hsp70-1/-4+GmPSS8 was generated using Agrobacterium-mediated methods.
Novel plant genotypes	T-DNA insertion mutants and their crossing lines were used in this study: hsp70-1 (SALK_135531), hsp70-4 and hsp70-1/-4.<br>hsp70-1/-4 was generated by crossing hsp70-1 with hsp70-4.
Authentication	The genotypes of hsp70-1/-4 were confirmed by genotyping using specific primers in F1 and F2 population.<br>GmPSS8 insertion in hsp70-1/-4 was confirmed genotyping using specific primers in T3 plants.
